# Understanding Barriers to Uptake of TB Preventive Treatment Among People Living with HIV in Zimbabwe: A Qualitative Assessment of Healthcare Workers’ Perspectives

**DOI:** 10.3390/tropicalmed11080225

**Published:** 2026-08-11

**Authors:** Tawanda Mapuranga, Collins Timire, Ronald T. Ncube, Sithabiso Dube, Nqobile Mlilo, Cynthia Chiteve, Owen Mugurungi, Tsitsi Mutasa-Apollo, Fungai Kavenga, Clorata Gwanzura, Manners Ncube, Nicholas Siziba, Selma Dar Berger, Talent Maphosa, Macarthur Charles, Riitta A. Dlodlo, Julia Ershova

**Affiliations:** 1 The Union Zimbabwe Trust, 261 Diamond Road, Westgate, Harare 00263, Zimbabwe; rncube@uzt.org.zw (R.T.N.); sdube@uzt.org.zw (S.D.); nmlilo@uzt.org.zw (N.M.); 2 AIDS and TB Department, Ministry of Health and Child Care, Harare 00263, Zimbabwe; collinstimire2005@yahoo.com (C.T.); mugurungi@gmail.com (O.M.); tsitsiapollo1@gmail.com (T.M.-A.); drkav8@gmail.com (F.K.); cloratag@gmail.com (C.G.); ncube.manners@gmail.com (M.N.); sitwalo@gmail.com (N.S.); 3 The International Union Against Tuberculosis and Lung Disease (The Union), 75001 Paris, France; cchiteve@uzt.org.zw (C.C.); selmadar@gmail.com (S.D.B.); rdlodlo@theunion.org (R.A.D.); 4 US Centers for Disease Control and Prevention, Harare 00263, Zimbabwe; oxv9@cdc.gov; 5 US Centers for Disease Control and Prevention, Atlanta, GA 30341, USA; xzk9@cdc.gov (M.C.); jhe3@cdc.gov (J.E.)

**Keywords:** TB preventive treatment, isoniazid preventive therapy, Zimbabwe

## Abstract

People living with HIV (PLHIV) are at an increased risk of progressing from *Mycobacterium tuberculosis* (TB) infection to TB disease. Tuberculosis preventive treatment (TPT) is recommended for PLHIV; however, its uptake remains suboptimal. In Zimbabwe, three months of weekly rifapentine and isoniazid (3HP) and six months of daily isoniazid (6H) are the most commonly prescribed TPT regimens. We explored barriers to TPT uptake among PLHIV from the perspective of healthcare workers (HCWs) in Zimbabwe. Facility nurses who had been implementing TPT for at least six months were invited to participate in in-depth interviews and/or focus group discussions conducted in March 2023. Audio recordings were transcribed into English, coded using NVivo 12 (QSR International), and analyzed thematically. Eleven HCWs participated in in-depth interviews and 15 in focus group discussions. Barriers were categorized as person- and health system-related. Person-related barriers included misconceptions about TPT, fear of adverse events, and pill burden. Health system barriers included stock-outs of TPT medications, limited HCW knowledge and skills to promote TPT, limited expertise in initiating TPT among children under five years of age, and limited access to chest radiography. Barriers to TPT uptake and completion remain in Zimbabwe and contribute to missed opportunities for TB prevention among PLHIV. TPT implementation could be strengthened through an uninterrupted supply of TPT medications, continuous training and mentorship of HCWs, and community engagement to improve awareness of the benefits and risks of TPT.

## 1. Introduction

Tuberculosis (TB) is an infectious disease caused by *Mycobacterium tuberculosis*, which is transmitted through the air when people with TB expel bacteria, for example, by coughing. Although TB is preventable and usually curable, more than 10 million people continue to develop TB each year, resulting in an estimated 1.23 million deaths in 2024 [1]. Approximately one-quarter of the world’s population is estimated to be infected with *M. tuberculosis* [2]. TB preventive treatment (TPT) substantially reduces the risk of progression from TB infection to TB disease and is up to 90% effective in preventing TB disease among people who complete treatment, making it a critical component of the WHO End TB Strategy [3]. People living with HIV (PLHIV) are at increased risk of progressing from *M. tuberculosis* infection to TB disease because of impaired cell-mediated immunity [1,4]. Globally, PLHIV are estimated to be approximately 18 times more likely to develop TB disease than people without HIV [4]. Although TPT has long been recommended for PLHIV, implementation remains suboptimal in many countries. In 2024, TPT coverage among PLHIV newly initiated on antiretroviral therapy (ART) was estimated at 58% worldwide [1].

Zimbabwe has a high burden of both HIV and TB, with an HIV/TB co-infection rate of 49%, an estimated TB incidence among PLHIV of 121 per 100,000 population, and an HIV prevalence of approximately 11% [1,5]. The Ministry of Health and Child Care (MOHCC) began implementing TPT for PLHIV in 2012 to reduce the burden of TB. In early 2019, the U.S. President’s Emergency Plan for AIDS Relief (PEPFAR) launched an initiative to accelerate TPT scale-up in Zimbabwe in support of the End TB Strategy targets. By September 2023, more than 891,000 PLHIV had completed TPT in PEPFAR-supported health facilities, representing 88.8% of PLHIV receiving ART in those facilities.

Effective and safe delivery of TPT requires implementation of a comprehensive cascade of care that includes identifying individuals at highest risk, screening for TB disease, testing for TB infection where indicated, selecting an appropriate preventive treatment regimen, managing adverse events, supporting treatment adherence, and monitoring program performance. Successful scale-up of TPT depends on identifying and addressing barriers to implementation. These barriers can be broadly categorized as health system- and person-related factors. Health system barriers include inadequate healthcare worker (HCW) knowledge of the benefits and risks of TPT, limited access to diagnostic tools to exclude TB disease, and stock-outs of TPT medicines and ancillary medications [6,7,8]. Person-related barriers include fear of adverse effects, inadequate knowledge about TPT, low motivation to initiate or complete treatment, pill burden, and contraindications to TPT, such as heavy alcohol use, liver disease, and drug–drug interactions [9,10,11]. Understanding these barriers is essential for developing context-specific strategies to optimize TPT uptake and completion.

In this study, we explored barriers to TPT uptake among PLHIV, including challenges for TPT initiation and completion, in selected PEPFAR-supported health facilities in Zimbabwe from the perspective of HCWs involved in TPT implementation. We also examined challenges related to excluding TB disease and explored misconceptions about TPT reported by HCWs. The findings will inform strategies to strengthen TPT implementation and improve TPT initiation, adherence, and completion among PLHIV, including children living with HIV (CLHIV), in Zimbabwe.

## 2. Methods

### 2.1. Study Design and Setting

We conducted a qualitative study using data collected as part of the “Evaluation and Optimization of Initiation, Adherence, and Completion of Tuberculosis Preventive Treatment (TPT) in PLHIV and Household Contacts of Newly Diagnosed People with TB in Zimbabwe” (ACE TPT) project. The ACE TPT project aimed to evaluate TPT initiation, adherence, completion, and adverse events among PLHIV and household contacts receiving TPT in 12 PEPFAR-supported health facilities in Zimbabwe during 2022–2023.

### 2.2. Study Population

Study participants were purposively selected to ensure representation from all 12 study health facilities and to capture a broad range of perspectives across different geographic locations and facility types, including primary healthcare clinics, district hospitals, provincial hospitals, and mission hospitals in rural and urban settings across eight districts in the three study provinces. Eligible participants were HCWs aged 18 years or older who had been involved in TPT implementation for at least six months. HCWs younger than 18 years, those who had been involved in TPT implementation for less than six months, and those not directly involved in TPT implementation activities were excluded from the study.

Sample size was guided by the principle of thematic saturation. Throughout data collection, the study team held regular debriefing meetings to review emerging findings and assess whether additional interviews generated new themes. Recruitment ceased when additional interviews yielded no substantively new themes, indicating that thematic saturation had been achieved.

Eligible HCWs participated in in-depth interviews (IDIs), followed by three focus group discussions (FGDs). The IDIs were conducted first to explore individual experiences, whereas the FGDs were subsequently used to validate and further explore the emerging themes through group discussion. All participating HCWs were nurses responsible for TB symptom screening, assessing eligibility for TPT, and initiating TPT during routine visits by PLHIV. They also conducted follow-up telephone calls to monitor treatment adherence, identify and report adverse events, and document treatment completion. Patients with suspected active TB or major adverse events were referred to medical officers for further evaluation and management.

### 2.3. Theoretical Framework

Health-related behaviors and decisions regarding the use of healthcare services are influenced by both individual characteristics and the social and environmental contexts in which people live and work. The Social Ecological Model (SEM) provides a framework for understanding how multiple levels of influence shape health behaviors and outcomes. In this study, the SEM was used to examine barriers and facilitators to TPT uptake and completion among PLHIV across four levels.

**Figure 1:**
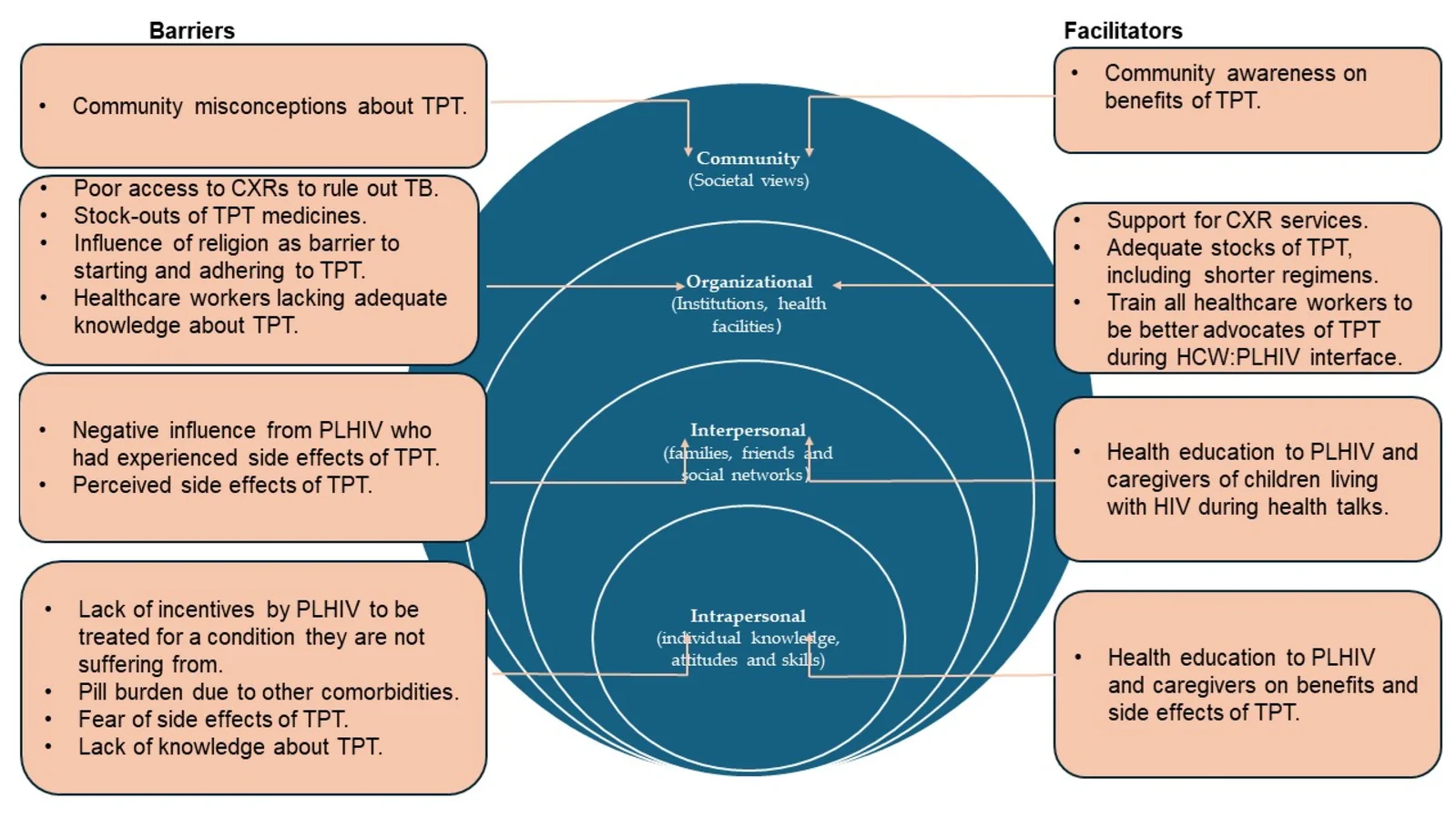
The Social Ecological Model.

The first level comprises intrapersonal (individual) factors, including knowledge, attitudes, and beliefs that influence decisions to initiate and complete TPT (Figure 1). The second level includes interpersonal factors, such as interactions with family members, friends, peers, and healthcare workers, which can shape health-related decisions and behaviors. The third level encompasses organizational or institutional factors. Informal institutions, such as religious organizations may discourage TPT initiation because of beliefs that preventive treatment reflects a lack of faith. Formal institutions, including health facilities, may influence TPT uptake and completion through the availability of diagnostic services to exclude TB disease, uninterrupted supplies of TPT medicines, and the knowledge, skills, and communication of healthcare workers. The fourth level comprises community factors, including prevailing social norms and community perceptions that may influence attitudes toward TPT and its acceptance.

The SEM recognizes that health behaviors and outcomes are shaped by interactions between individual (intrapersonal) and broader interpersonal, organizational, and community influences, many of which are beyond an individual’s direct control [12,13]. We applied the SEM as a conceptual framework to understand barriers and facilitators to TPT uptake and completion among PLHIV.

### 2.4. Data Collection and Management

An interview guide was developed and piloted through interviews with two HCWs recruited from a non-participating health facility (Supplementary Materials, S1). Findings from the pilot interviews were used to refine the interview and FGD guides but were not included in the analysis. The interviews were conducted in March 2023 by members of the study team and led by a PhD student with a Master of Science (MSc) degree who was trained in qualitative research methods. Each interview lasted 25–35 min and was conducted in Shona or a combination of Shona and English, according to participants’ preferences.

The interview guide explored the following themes: challenges and facilitators of TPT implementation; perceived barriers to TPT initiation and adherence; challenges related to TPT documentation; and strategies used by HCWs to ensure the uninterrupted availability of TPT medicines. These themes were informed by published literature and the research team’s experience with TPT implementation in Zimbabwe. Following each interview, the research team held debriefing meetings to discuss the findings and identify emerging themes to be explored in subsequent interviews (Supplementary Materials, S2). This iterative process continued until data saturation was reached, defined as the point at which no new information or themes emerged [14].

All HCWs who participated in the IDIs were invited to participate in one of three FGDs, with one discussion conducted in each of the three study provinces (Mashonaland East, Mashonaland West, and Mashonaland Central). HCWs who had not participated in the interviews were also invited to participate in the FGDs. Because only a limited number of HCWs at each facility were directly involved in TPT implementation and met the eligibility criteria, many participants contributed to both the IDIs and FGDs. The FGDs were conducted after completion of the IDIs to validate, refine, and further explore the themes that had emerged during the interviews through group discussion. The FGDs were conducted after completion of the IDIs to validate, refine, and further explore the themes that had emerged during the interviews through group discussion. The discussions were facilitated by two members of the research team and lasted 35–40 min. Following each FGD, the research team met to discuss the findings and identify issues requiring further exploration in subsequent discussions.

All interviews and FGDs were audio-recorded and conducted in private locations within the participating health facilities, as preferred by the participants. Audio recordings were stored securely in encrypted environments and destroyed after transcription. Participants received USD10 as compensation for their time, in addition to reimbursement of transportation costs, which was determined on a case-by-case basis.

### 2.5. Data Analysis

All audio recordings were transcribed into Microsoft Word in Shona and subsequently translated into English by the same individual. To ensure the accuracy of the translations, the translator listened to the original audio recordings while reviewing the English transcripts. The translated transcripts were imported into NVivo 12 (QSR International) for data management and coding by the study data manager. Data were analyzed thematically following the approach described by Braun and Clarke (2006) [15]. Transcripts from the IDIs and FGDs were analyzed together, with the FGDs serving to triangulate, validate, and enrich the interpretation of themes emerging from the interviews rather than to provide independent participant counts.

The transcripts were first read repeatedly to achieve familiarization with the data. Before coding, two researchers independently reviewed four interview transcripts to identify preliminary codes, sub-themes, and themes. They subsequently held a debriefing session to compare their initial coding, discuss discrepancies, and reach consensus. The resulting coding framework was reviewed by the study team before being applied to the remaining transcripts. Coding combined both deductive and inductive approaches. Deductive codes were informed by the literature, the research team’s experience, the interview guide, and the SEM, while inductive codes emerged directly from the data. Related codes were grouped into sub-themes, which were subsequently organized into overarching themes. Additional debriefing sessions were held to refine, define, and name the themes. Finally, the themes were categorized as barriers or facilitators to TPT uptake and adherence among PLHIV, and relationships among themes were explored to develop a thematic map. All transcripts were de-identified prior to analysis.

### 2.6. Ethics

Ethics approval was obtained from the Medical Research Council of Zimbabwe (MRCZ/A/2746) and classified as research. All the participants gave written informed consent to be audio-recorded and to take part in the qualitative interviews and their right to withdraw from the study at any time without penalty. This activity was reviewed by the US Centers for Disease Control and Prevention (CDC) and deemed not research but rather a program evaluation within public health practice (U.S. law).

## 3. Results

Overall, 16 HCWs participated in the study. Eleven HCWs from 11 health facilities participated in the in-depth interviews, 10 of whom also participated in the focus group discussions (FGDs). An additional five HCWs participated only in the FGDs, bringing the total number of health facilities represented in the FGDs to 12. Among the 11 HCWs who participated in the interviews, seven (64%) were female. Their mean duration of TPT implementation experience was 4 years (range: 1–8 years), and their mean duration of service was 16 years (range: 6–30 years). Similar characteristics were observed among the 15 HCWs who participated in the FGDs (Table 1).

Three overarching themes emerged from the analysis of the collected data: (1) barriers to TPT uptake and completion at the intrapersonal, interpersonal, organizational, and community levels; (2) facilitators and measures to support TPT completion; and (3) healthcare workers’ recommendations for improving TPT implementation.

### 3.1. Barriers to TPT Uptake and Completion

HCWs were asked to describe barriers to TPT implementation in their health facilities, including challenges related to TB symptom screening, TPT initiation, patient follow-up, and documentation of treatment outcomes. The barriers to TPT uptake and completion identified by HCWs were categorized and summarized in Table 2 according to the SEM into four levels: intrapersonal, interpersonal, organizational, and community.

#### 3.1.1. Intrapersonal Barriers

The intrapersonal barriers reported by HCWs included pill burden, fear of TPT-related side effects, limited knowledge about TPT, and lack of motivation to take preventive treatment for a condition in the absence of symptoms.

According to HCWs, some PLHIV openly declined TPT because of concerns about pill burden or fear of side effects based on the experiences of others. Others accepted the medication during clinic visits but later discarded it before returning home.

*…Thus, it was difficult for them to refuse to take the medicines while at the facility, but there are some who would say, ‘Do not give me those ones. I do not want them.’ Then some may take them, but later on throw the medicines away. There is an open space which was full of boxes of TPT medicines that were strewn all over.*

Healthcare workers reported that PLHIV whose previous course of isoniazid preventive therapy had been interrupted because of medicine stock-outs were often reluctant to restart TPT. According to one HCW, this experience led some PLHIV to perceive that healthcare workers were not committed to their care, reducing their trust in the program. In addition, because these individuals had not completed TPT yet had not subsequently developed TB disease, some questioned the value of restarting or completing preventive treatment.

*…A patient would get a 3 month’s supply and when they come back for re-supplies, the medicines would be out of stock. That patient would have defaulted and may not enjoy the benefits of the medicines. We had to start them again. Our clients did not like that. For them it seems like we were joking with their health. They argue why they should take TPT medicines, yet they are not sick and have not suffered from TB before.*

HCWs reported that PLHIV initiating ART were more likely to accept TPT than those who had been on ART for a longer period. According to HCWs, patients receiving long-term ART were less willing to initiate TPT because they believed that improvements in their immune function reduced their risk of developing TB.

*…I observed that TPT is well accepted if initiated within a month or 3 months of starting ART, though not on the day they start ART. PLHIV who have been in the system [on ART] for a long time and delayed starting TPT consider themselves to have improved immune systems.*

#### 3.1.2. Interpersonal Barriers

HCWs reported that misconceptions about TPT were common and were often reinforced through misinformation shared by other PLHIV, family members, or people within the community. As one HCW explained

*…Then there are those who are misinformed by other people, and they stop taking their medicines.*

Fear of TPT-related side effects was another commonly reported interpersonal barrier to treatment completion. Reports of adverse events experienced by other PLHIV, particularly skin rashes, blisters, and peripheral neuropathy associated with isoniazid, discouraged some individuals from initiating TPT. In addition, some PLHIV who experienced minor side effects after starting TPT discontinued treatment because they feared developing more severe adverse events.

*…One may discourage others from taking TPT. For some it is due to pill burden while some may develop itchiness, and they stop taking the medicines. That is what they say.*

The absence of symptoms reduced some patients’ motivation to initiate TPT, as they perceived little benefit in taking preventive treatment for a condition they did not believe they had.

*…One patient can actually say, ‘No, HIV is the one I got tested for’ and ‘Yes, I am positive and that is the reason why I am taking ART. This medicine you are giving me before I get sick with TB … you will treat me when I get sick with TB.’*

#### 3.1.3. Community Barriers

Healthcare workers reported that community beliefs and misinformation often influenced PLHIV’s decisions about TPT more strongly than the information provided by HCWs during health education sessions, affecting both TPT uptake and adherence.

*…In their communities, there are some who call themselves nurses and doctors. They [PLHIV] bring the information they get from such people to clinics. They tell us ‘We heard others saying this about the medicines and for this reason I don’t want to take them.*

#### 3.1.4. Organizational/Institutional Barriers

Organizational and institutional barriers arose from both formal and informal institutions. Among the informal institutions, HCWs identified religious beliefs, particularly those associated with the Apostolic faith, as an important barrier to TPT uptake and adherence. According to HCWs, the influence of these beliefs often outweighed the health education provided at health facilities, and taking TPT was perceived by some followers as a sign of weak faith.

*…Some people have their belief systems, they go to churches where they are told if you take such medicines, you do not have strong faith in God.*

In contrast, formal institutions, including clinics and hospitals, played a pivotal role in influencing both TPT uptake and adherence. The most commonly reported organizational barriers included stock-outs of TPT medicines, poor communication between clinic and pharmacy staff, limited access to or the high cost of chest X-ray (CXR) services excluding TB disease, and inadequate knowledge among HCWs resulting from insufficient training on TPT. Consequently, some HCWs reported hesitancy to initiate TPT in specific population groups, including young children (<5 years), adolescents and highly educated individuals, older adults, and PLHIV who had already been receiving ART.

Stock-outs/shortage of TPT and ancillary medicines was the most common barrier for not initiating and completing TPT course.

*…We used to give the patient 3 months’ supplies and when they came back for a re-supply from our pharmacy, they would be told that the medicines were out of stock.*

*…The challenge we used to encounter was that a patient would get a 3 month’s supply [of 6H] and when they come back for re-supplies, the medicines would be out of stock.*

Poor communication between clinic and pharmacy staff was observed during the early stages of TPT implementation. Inadequate coordination contributed to stock-outs of TPT medicines, forcing some PLHIV to interrupt treatment. Pharmacy staff had not received training on TPT implementation, and communication with clinical staff regarding forecasting medicine needs and maintaining adequate supplies for both new and existing TPT patients was reported to be insufficient.

*…I once encountered that challenge when I was dispensing INH. We used to give 3 months’ supplies to patients and when they came back for re-supplies from our pharmacy, they would be told that the medicines were out of stock.*

HCWs reported limited access to chest X-ray (CXR) services as a barrier to TPT initiation. In some health facilities, CXR services were unavailable because of frequent power outages, while in others they were outsourced to private providers, making them unaffordable for many patients. As a result, the limited availability and high cost of CXR services hindered efforts to exclude TB disease before initiating TPT.

*…We are now able to carry out tests, such as GeneXpert on sputum though we still have challenges in accessing CXRs, and we also experience power outages for long periods. As such, we encourage people to have CXR done elsewhere so that the doctor will review them. Usually, they are charged USD20 and many cannot afford that.*

*…The hospital does not have X-ray services, so we refer patients to private [facilities]. Some of the patients cannot afford X-ray services. As such, regarding TB screening, the challenge is X-ray services, but we have a lab to test sputum specimens.*

HCWs observed that the number of staff trained to implement TPT was often insufficient. In addition, routine staff rotations within health facilities meant that trained HCWs were frequently reassigned to other departments, leaving TPT services to be delivered by untrained staff. As a result, HCWs who had not received TPT training were perceived to have limited knowledge of TPT and were less likely to take ownership of TPT implementation.

*…if few people are trained, those who are not trained may end up saying, ‘This is not for us.’ You will notice that during the days when trained staff are not on duty, very few patients are initiated on TPT because the untrained staff may not be knowledgeable enough.*

Some HCWs expressed hesitancy to initiate TPT in children living with HIV (CLHIV). This was attributed to the lack of pediatric formulations and limited experience in initiating and managing TPT in children younger than 5 years. In addition, HCWs reported that TPT uptake among CLHIV was challenging because of the perceived increase in pill burden, as children were usually prescribed daily isoniazid for six months (6H), and the need to obtain consent from their caregivers before.

*…I still need some assistance on starting pediatrics under five years, otherwise I am confident to start adults on TPT…the pharmacy may not have their formulations. Children are usually given packages [Fixed Dose Combinations].*

*…Children are challenging to give [TPT]. They need adequate explanation. They are young and they do not understand these things. They stay with caregivers. They usually have their medicines and if you add another type of medicines, children ask a lot of questions.*

According to HCWs, educated individuals, adolescents, and caregivers of children living with HIV (CLHIV) often asked detailed questions about TPT before deciding whether to initiate treatment. HCWs reported that TPT uptake among these groups depended largely on whether their questions were answered satisfactorily and misconceptions were addressed. Some HCWs, particularly those who had not received TPT training, acknowledged that they lacked sufficient knowledge to confidently counsel and advocate for TPT, resulting in missed opportunities for treatment initiation.

*…We do not have adequate information regarding TPT. Thus, we struggle to market TPT since we have limited information about it. So maybe there is a need to empower health workers with adequate information about TPT.*

*…So, the patient may be wanting to take TPT but the way the product [TPT] is marketed gives him doubt.*

Patients’ adherence to ART was commonly used by HCWs to judge their likely adherence to TPT. Consequently, some HCWs expressed hesitancy to initiate TPT in adolescents and older adults because they perceived these groups to be at greater risk of poor treatment adherence. Adherence among adolescents was considered particularly challenging during school terms, when many attended boarding schools and lacked support from their parents or caregivers.

Non-disclosure of HIV status by parents or caregivers was also identified as a barrier to adherence to both ART and TPT among children and adolescents. This included non-disclosure to the children themselves and, for adolescents attending boarding schools, to key individuals such as school health staff, thereby limiting the support available to promote treatment adherence.

*…If they go to a boarding school for 3 months, some of them do not take their medicines. By the time they go on holiday to collect their supplies…if you collect a [blood] sample, you will notice the VL will be very high…So it is a challenge for us to give them TPT while they are not complying with ART. If you add [TPT] to them whilst they have ART, their compliance with medication is a challenge because we already have challenges with adolescents adhering to [ART] medicines.*

By contrast, HCWs observed that the older adults with multiple comorbidities were less likely to adhere to TPT due to pill burden.

*…Those who I feel their compliance may be a challenge. The elderly, those above 60 years…because of pill burden. Some will be on DM [diabetes mellitus medicines] and might also be on HPT [hypertension medicines] as well as on ART.*

HCWs attributed poor adherence to TPT among children to the use of six months of daily isoniazid (6H) rather than the shorter 3HP regimen. They explained that the longer treatment duration, combined with pill burden, reduced adherence, particularly among adolescents.

*…In most cases we give 6H to children, so the duration is long.*

### 3.2. Facilitators/Measures to Support TPT Completion Among PLHIV

Despite the barriers described above, HCWs identified several measures that helped improve TPT completion among PLHIV. These measures included effective coordination between HIV clinic and pharmacy staff to maintain an uninterrupted supply of TPT medicines, together with other strategies aimed at supporting treatment adherence and completion.

*…We manage our stocks, and we work together with people who order medicines. Whenever our stocks are below critical level, we place an emergency order.*

*…Thus, we discussed with staff from the pharmacy and agreed to set aside a 3 month’s supply of TPT for those we would have given 3 months’ supplies.*

While almost all facilities dispensed the full course of 3HP to eligible PLHIV at treatment initiation, different approaches were used to ensure completion of the six-month isoniazid (6H) regimen. In most facilities, PLHIV received an initial three-month supply of 6H, while the remaining three-month supply was reserved for the same patient and dispensed when they returned for follow-up.

*…We give them a once-off supply. If it is 3HP, we give a supply for 12 weeks.*

*…We usually give 3 months’ supplies of isoniazid, and when the patient comes back, we give another 3 months’ supply. Even this one which is taken for 3 months [3HP regimen], we give a full supply and the patient goes away.*

### 3.3. Recommendations Suggested by HCWs to Improve TPT Implementation

Based on their experience with TPT implementation, HCWs proposed several recommendations to improve TPT uptake and completion. The most frequently suggested recommendation was to strengthen education for both PLHIV and HCWs. In particular, HCWs emphasized the importance of integrating TPT education into routine health talks delivered at health facilities to improve patients’ understanding of TPT and encourage both treatment initiation and completion.

*…People must understand why they are taking TPT. The side effects must be talked about as well. Patients need adequate information so that even if they experience side effects, they know these were mentioned. They know it is not a major problem, and they have to continue taking their medicines. What is critical is to empower patients with knowledge.*

*…I think health education helps. Whenever we do health talks in the morning, we need to include TPT.*

Capacity building of HCWs was identified as a key requirement for successful TPT implementation. Training sufficient numbers of HCWs was considered essential to ensure effective implementation of TPT. Participants also emphasized the importance of including staff from all relevant departments within health facilities to ensure a shared understanding of the purpose, benefits, and risks of TPT and to promote coordinated implementation across services.

*…Then when the program starts…. maybe, almost everyone should be trained.*

Ensuring an uninterrupted supply of TPT medicines was the second major recommendation made by HCWs. Participants reported that the shorter 3HP regimen was generally better accepted than 6H and contributed to improved TPT uptake and adherence. However, some health facilities experienced stock-outs or irregular supplies of 3HP during the implementation period. HCWs therefore emphasized the need to maintain an uninterrupted supply of shorter TPT regimens to support treatment initiation, adherence, and completion.

*…From the moment we started giving out 3HP, uptake of TPT increased compared to previous years.*

*...We are appealing for 3HP because patients usually complain about pill burden especially due to isoniazid. Isn’t INH taken daily for 6 months? But we are requesting for those that are taken once per week. It helps. We only have INH. We got a few quantities of 3HP this week, but the stocks are finished already.*

## 4. Discussion

In this study we aimed to identify barriers to TPT uptake among PLHIV in selected PEPFAR-supported health facilities to inform strategies for strengthening TPT implementation in Zimbabwe. Barriers to TPT initiation and adherence included intra-/interpersonal factors (fear of side effects) and organizational/institutional factors (stock-outs of TPT medicines, influence of informal religious organizations that are against use of healthcare services, limited experience among HCWs in initiating TPT in CLHIV, and inadequate knowledge among HCWs about the benefits and risks of TPT) and community factors (misconceptions about TPT).

Lack of adequate knowledge about TPT among HCWs and PLHIV’s perception of HCWs as experts responsible for determining the appropriate TPT regimen were identified as important intra-/interpersonal factors. While effective HCW–PLHIV interactions improve patient satisfaction and TPT uptake, ineffective interactions may lead to stress, mistrust, and reduced cooperation [16,17]. Beyond interpersonal interactions, HCWs’ knowledge and experience with TPT—both of which are assessed by PLHIV during clinical encounters—play a critical role in determining TPT uptake. HCWs should clearly and confidently explain the benefits and risks of TPT and why preventive treatment is recommended even for PLHIV who do not have symptoms of TB. These discussions should take place in an environment where PLHIV feel respected, are treated with dignity, and have their concerns addressed [18]. Accordingly, HCWs’ knowledge, confidence, and communication skills are essential for improving both TPT uptake and adherence [6]. HCWs who are well informed about TPT can serve as confident advocates, educating PLHIV about its benefits and addressing concerns about treatment. Conversely, HCWs with limited knowledge may inadvertently create doubt and perpetuate misconceptions about the purpose and effectiveness of TPT [7,10]. PLHIV who remain unconvinced of the benefits of TPT may subsequently discontinue treatment or seek advice from peers or community members, potentially reinforcing misinformation, as observed in this study.

Organizational/institutional factors, such as stock-outs of TPT medicines have been described by researchers in Nigeria, India and Uganda [10,19,20,21]. Firstly, stock-outs of TPT medicines, including pyridoxine, were reported by all respondents, to varying degrees, and resulted in missed opportunities for TPT uptake. Moreover, some PLHIV receiving ART who had previously been unable to complete TPT because of medicine stock-outs subsequently declined re-initiation of TPT, suggesting that treatment interruptions attributable to health system factors may negatively influence patients’ willingness to restart and complete preventive treatment. This interplay of intrapersonal, interpersonal and organizational factors negatively impacts uptake and adherence to TPT. Effective TPT implementation requires sustained and uninterrupted availability of TPT medicines, particularly shorter rifamycin-based regimens where recommended, together with reliable procurement and supply chain systems to prevent stock-outs.

Increasing demand for TPT among PLHIV will require addressing barriers to TPT uptake at multiple levels, particularly among adults already receiving ART and caregivers of CLHIV, through education on the benefits of preventive treatment before the onset of TB symptoms [22]. Previous studies from Ethiopia, Cambodia, and South Africa have similarly shown that fear of side effects and limited knowledge about TPT among parents and caregivers contribute to low TPT uptake among eligible children [20,23,24,25]. At the organizational level, inadequate knowledge of TPT among HCWs has also been identified as a barrier to TPT uptake in South Africa and Zambia [11,26]. Consistent with these findings, some HCWs in our study expressed hesitancy to initiate TPT in CLHIV younger than 5 years, suggesting that insufficient training may reduce healthcare workers’ confidence in counseling caregivers and initiating preventive treatment. Overall, our findings demonstrate that intrapersonal, interpersonal, organizational, and community-level factors interact to influence both TPT uptake and adherence, underscoring the need for comprehensive interventions that address barriers across multiple levels of the Social Ecological Model.

Although this study was conducted in PEPFAR-supported health facilities in Zimbabwe, many of the barriers identified are likely to be relevant to TB preventive treatment implementation in other high-TB and high-HIV-burden settings. Misconceptions about TPT, concerns about adverse events, inadequate healthcare worker knowledge, and interruptions in medicine supply have been reported in a wide range of resource-limited settings, suggesting that these challenges are not unique to Zimbabwe. However, the relative importance of specific barriers may vary depending on health system organization, the availability of trained healthcare workers, access to diagnostics and medicines, and the level of integration between TB and HIV services. Strengthening healthcare worker training and mentorship, ensuring uninterrupted availability of TPT medicines, providing consistent patient education, and integrating TPT into routine HIV care are likely to improve TPT uptake across diverse healthcare settings, although implementation strategies should be tailored to the local context and available resources.

The findings also demonstrate that barriers to TPT uptake operate at multiple levels, including the individual, interpersonal, and health system levels. Consequently, interventions focusing solely on patient education or healthcare worker training are unlikely to achieve sustained improvements in TPT uptake unless accompanied by broader health system strengthening measures, including uninterrupted medicine supply, supportive supervision, and integration of TB preventive services into routine HIV care.

Our study has several limitations. First, the same HCWs participated in both the in-depth interviews and focus group discussions because only a limited number of HCWs were involved in TPT implementation at each facility. Although this may have reduced the diversity of perspectives, triangulation of findings from both data collection methods enhanced the credibility of the results. Second, PLHIV were not interviewed because of implementation time constraints. Consequently, the findings reflect HCWs’ perspectives on barriers to TPT uptake and completion, rather than the direct experiences of PLHIV. However, purposive selection of HCWs with at least six months of experience implementing TPT provided rich insights into patient- and health system-related barriers. Finally, the study was conducted exclusively in PEPFAR-supported health facilities; therefore, the findings may not be generalizable to health facilities in Zimbabwe that do not receive PEPFAR support.

## 5. Conclusions

This study identified important barriers to TPT uptake and completion among PLHIV receiving care in PEPFAR-supported health facilities in Zimbabwe, a country with a high burden of HIV and TB. Addressing these barriers is critical to improving TPT coverage and reducing the burden of TB among PLHIV. An uninterrupted supply of TPT medications could reduce missed opportunities for treatment initiation caused by drug stock-outs. TPT implementation could also be strengthened through continuous training and mentorship of HCWs on platforms such as TB ECHO sessions. In addition, religious and other sociocultural barriers to TPT uptake and completion may be addressed through community engagement and awareness campaigns to improve understanding of the benefits of TPT.

## Figures and Tables

**Table 1:** Characteristics of HCWs who participated in in-depth interviews and focus group discussions.

Type of Interview	Sex	n (%)	Work Experience in Years (Mean, Range)	
			As a HCW (Nurse)	Implementing TPT
Interviews, n = 11	Male	4 (36%)	12 (3–24)	6 (1–10)
	Female	7 (64%)	16 (6–30)	4 (1–8)
FGDs, n = 15	Male	4 (27%)	12 (3–24)	6 (1–10)
	Female	11 (73%)	15 (6–30)	3.5 (1–8)

HCW = Healthcare worker; TPT = Tuberculosis preventive treatment.

**Table 2:** Barriers to starting and completing TPT.

Associated Factor	Barrier	Quote
Intra-personal/interpersonal factors	Misconceptions about TPT	That person [PLHIV] would inform others that the medicines are not good... HCW-04
	Pill burden	Some refuse because of pill burden. Some who are taking TPT medicines are also suffering from blood sugar or blood pressure. Thus, they end up saying the number of pills they are taking is too much. HCW-06
	Fear of side effects of TPT	One may discourage others to take TPT. For some it is due to pill burden while some may develop itchiness, and they stop taking the medicines. That is what they say. HCW-08 …the other barriers are that patients whose relatives once developed side effects to certain TPT medicines would refuse. So, the patients were afraid of the unknown and they think they may also experience the same reactions. HCW-01
	Lack of incentives to be treated for a condition one is not suffering from	One patient can actually say, ‘No, HIV is the one I got tested for and ‘Yes, I am positive and that is the reason why I am taking ART. This medicine you are giving me before I get sick with TB… you will treat me when I get sick with TB.’ HCW-04
Organizational/institutional factors	Shortage of TPT and ancillary medicines	We are not stocked most of the time. If we get supplies, they are usually not enough such that we give to a few people. In most cases the stocks do not last a month. Looking at the number of PLHIV we serve in our facility; it’s almost 2000. HCW-04
	Hesitancy by HCW to start children under 5 years on TPT	I still need some assistance on starting pediatrics under five years, otherwise I am confident to start adults on TPT…the pharmacy may not have their formulations. HCW-07
	Lack of skills to advocate for TPT among HCWs	…So, the patient may be wanting to take TPT but the way the product [TPT] is marketed gives him doubt.
	Lack of mechanisms to protect TPT course of PLHIV	We used to give the patient 3 months supplies and when they came back for a re-supply from our pharmacy, they would be told that the medicines were out of stock. HCW-08 The challenge we used to encounter was that a patient would get a 3 month’s supply [of 6H] and when they come back for re-supplies, the medicines would be out of stock. HCW-07
	Unavailability of CXR services to rule out active TB	The hospital does not have X-ray services, so we refer patients to private [facilities]. Some of the patients cannot afford X-ray services. As such, regarding TB screening, the challenge is X-ray services, but we have a lab to test sputum specimens. HCW-04
	Religious doctrine acting as a barrier to starting TPT	Some people have their belief systems, they go to churches where they are told if you take such medicines, you do not have strong faith in God. HCW-02
Community-level factors	Influence of people within communities	Then there are those who are misinformed by other people, and they stop taking their medicines.

## Data Availability

The data presented in this study are available on request from the corresponding author. The data are not publicly available due to authorizations that may be required by the funder.

## Supplementary Materials

The following supporting information can be downloaded at https://www.mdpi.com/article/10.3390/tropicalmed11080225/s1, Supplementary Materials S1: Healthcare worker interview guide; Supplementary Materials S2: Healthcare worker focus group discussion guide.
