# Supplementary material for: Understanding Barriers to Uptake of TB Preventive Treatment Among People Living with HIV in Zimbabwe: A Qualitative Assessment of Healthcare Workers’ Perspectives
Source: Trop Med Infect Dis. 2026 Aug 11;11(8):225. doi: 10.3390/tropicalmed11080225 (PMC13517091; doi:10.3390/tropicalmed11080225)
Supplement: Supplementary file 1 [file tropicalmed-11-00225-s001.zip › tropicalmed-4348755-supplementary.pdf]

Supplementary Materials, S1: Healthcare worker interview guide

Evaluation of tuberculosis preventive treatment (TPT) initiation, adherence and completion, and frequency and severity of adverse events associated with TPT in PLHIV, including CLHIV, in Zimbabwe

Principal Investigator: Ronald Ncube, [*M.D. MPH*]
Phone number: +263 782706866

Interview Guide: HCWs providing TPT

Based on typical qualitative approaches, some questions that do not appear on this list but are of a similar scope as the questions presented here may be included if relevant to the course of the interview. In addition, not all questions will be asked of all participants. Questions with prior are used to probe when relevant.

Introductory Script: Good morning/afternoon/evening thank you so much for participating in this interview. I am very interested in learning about your experiences with TPT programming. I am also interested in ideas that you might have about how to improve TPT initiation and completion. Before we begin, I would like to remind you that participation in this interview is voluntary and anything you say will be kept confidential. With your permission your responses will be audio recorded, however they will only be shared with other people as part of a summary report, with no names or other identifying information. You can stop the interview at any point or skip questions you would prefer not to answer. Before we get started, do you have any questions for me?

Part I: Introductory Questions

1. How are you today?

2. If this interview extends for a long period of time, would you be willing to return at a different point in time to finish the interview?

Transition Script: Ok let's get started. To begin, I am going to ask you about your understanding around the TPT program.

Part II: Process

1. ***[Opinions]*** *What is going well in the TPT services in this facility? What can be improved?*

2. *What is your role in providing TPT in this facility / in the HIV/ART clinic here? Walk through from start to finish how TPT is implemented.*

   [Eligibility] *Who is recommended to receive TPT? Is TPT routinely enquired about in each patient visit?*

   [Exclusion] *What criteria may exclude people from TPT? Which patients should not be offered TPT because of increased risk for adverse effects?*

   How confident are you initiating TPT?

3. ***[Monitoring during]*** *How long should TPT be continued (describe by regime)? Please describe how adverse event monitoring is performed in this facility / at TPT follow-up.*

4. ***[Monitoring post]*** *Please describe how adverse event/breakthrough TB monitoring is performed after TPT completion.*

Part III: Risks & Benefits
Transition Script: Next, I am going to ask you about your understanding of TPT.

5. ***[Why TPT?]*** *Please describe your understanding of the rationale of TPT / why TPT should be taken?*

   What are the risks?

   What are the benefits?

   What are your personal beliefs and feelings about TPT?

Part IV: Barriers
Transition Script: Next, I want to understand your perceptions of barriers or challenges to TPT service delivery.

6. ***[Barriers to initiation]*** *What are the challenges to provision of TPT in this facility / initiation?*

   What are the barriers to identifying and initiating TPT in eligible patients?

   Are there challenges in obtaining medicines to start TPT? If Yes, what are the challenges?

   Are there eligible patients hesitant to start TPT?

   Are you hesitant to start certain patients on TPT?

   How frequently do TPT medicine (including pyridoxine) stock outs occur?

7. ***[Barriers to adherence]*** *What are the barriers to TPT adherence?*

   Which mechanisms in place to ensure that TPT medicines are available once a patient starts TPT (protecting course for TPT)? Are there challenges in obtaining TPT medicines once a patient has started?

   Are there challenges in documenting TPT follow-up/completion?

   What are the most common reasons why patients in the catchment area of this facility do not complete TPT after they have started it?

   How do you think adherence to TPT can be improved?

Closing Script: Thank you so much for your participation in this interview. If you want to talk more about this or you have additional questions for me, please feel free to reach out (share contact information if interviewee is interested).

Supplementary Materials, S2: Healthcare worker focus group discussion guide

Evaluation of tuberculosis preventive treatment (TPT) initiation, adherence and completion, and frequency and severity of adverse events associated with TPT in PLHIV, including CLHIV, in Zimbabwe (ACE TPT)

Principal Investigator: Ronald Ncube, [*M.D. MPH*]
Phone number: +263 782706866

Focus Group Discussion Guide: HCWs providing TPT

Based on typical qualitative approaches, some questions that do not appear on this list but are of a similar scope as the questions presented here may be included if relevant to the course of the focus group discussion. In addition, not all questions will be asked of all participants. Questions with prior are used to probe when relevant.

Introductory Script: Good morning/afternoon/evening thank you so much for participating in this focus group discussion. I am very interested in learning about your experiences with TPT programming. I am also interested in ideas that you might have about how to improve TPT initiation and completion. Before we begin, I would like to remind you that participation is voluntary and anything you say will be kept confidential. With your permission your responses will documented via notes, however they will only be shared with other people as part of a summary report, with no names or other identifying information. You can leave this discussion at any point. Before we get started, do you have any questions for me? Ok let's get started. To begin, I am going to ask you about your understanding around the TPT program.

Part I: Process

4. ***[Opinions]*** *What is going well in the TPT services in this facility? What can be improved?*

5. *Walk through from start to finish how TPT is implemented.*

   [Eligibility] *Who is recommended to receive TPT? Is TPT routinely enquired about in each patient visit?*

   [Exclusion] *What criteria may exclude people from TPT? Which patients should not be offered TPT because of increased risk for adverse effects?*

   How confident are you initiating TPT?

6. ***[Monitoring during]*** *How long should TPT be continued (describe by regime)? Please describe how adverse event monitoring is performed in this facility / at TPT follow-up.*

5. ***[Monitoring post]*** *Please describe how adverse event/breakthrough TB monitoring is performed after TPT completion.*

Part II: Risks & Benefits

Transition Script: Next, I am going to ask you about your understanding of TPT.

8. ***[Why TPT?]*** *Why TPT should be taken?*

   What are the risks?

What are the benefits?

What are your personal beliefs and feelings about TPT?

Part III: Barriers
Transition Script: Next, I want to understand your perceptions of barriers or challenges to TPT service delivery.

9. ***[Barriers to initiation]*** *What are the challenges to provision of TPT in this facility / initiation?*

    What are the barriers to identifying and initiating TPT in eligible patients?

    Are there challenges in obtaining medicines for starting TPT?

    Are there eligible patients who may be hesitant to start TPT?

    Are you hesitant to start certain patients on TPT?

    How frequently do stock outs of TPT medicine (including pyridoxine) occur ?

10. ***[Barriers to adherence]*** *What are the barriers to TPT adherence?*

    How do you ensure that TPT courses for patients are protected? Are there challenges in obtaining TPT medicines once a patient has started?

    Are there challenges in documenting TPT initiation, follow up and completion? (one stage at a time)

    What are the most common reasons why patients in the catchment area of this facility do not complete TPT after they have started it?

    How do you think adherence to TPT can be improved?

Closing Script: Thank you so much for your participation in this focus group discussion. If you want to talk more about this or you have additional questions for me, please feel free to reach out (share contact information if participant is interested).
